# Meteorological Diary, Kept at Clapton, Hackney, from March 27 to April 24, 1810

**Published:** 1810-08

**Authors:** Thomas Foster


### *Meteorological Diary, kept at Clapton, Hackney, from March 27 to April 24, 1810, by* THOMAS FOSTER, *Esq.*

| Day | | Therm. Max. | Therm. Min. | Barometer. Max. | Barometer. Min. | Wind. | Weather. |
|---|---|---|---|---|---|---|---|
| March | 27 | 51° | 41° | 29 80 | 29 65 | S. S. E. | Clouds & rain |
| ☾ | 28 | 52 | 35 | 29 94 | 29 89 | W. | Sun & showers |
| | 29 | 55 | 34 | 29 99 | 29 95 | W. | Clear & clouds |
| | 30 | 54 | 41 | 29 96 | 29 80 | S. W. | Fair day |
| | 31 | 51 | 39 | 29 80 | 29 49 | S. | Fair—rain |
| April | 1 | 51 | 41 | 29 72 | 29 55 | S. E. | Fair |
| | 2 | 48 | 42 | 29 80 | 29 67 | E. | Small rain—fair |
| | 3 | 57 | 40 | 29 67 | 29 41 | S. W. | Small rain |
| ● | 4 | 49 | 33 | 29 77 | 29 45 | N.—S. | Showers |
| | 5 | 51 | 43 | 29 67 | 29 49 | S. W. | Clear—cloudy |
| | 6 | 42 | 41 | 29 34 | 29 33 | S. | Sun & showers |
| | 7 | 52 | 38 | 29 51 | 29 40 | S. | Sun and showers |
| | 8 | 54 | 42 | 29 52 | 29 50 | S. W. | Fair, *a. m.*—rain, *p. m.* |
| | 9 | 51 | 42 | 29 56 | 29 48 | S. E. | Rainy |
| | 10 | 45 | 33 | 29 70 | 29 66 | N. | Rainy |
| ☽ | 11 | 43 | 31 | 29 92 | 29 72 | N. E. | Showers |
| | 12 | 40 | 29 | 29 95 | 29 95 | N. | Fair |
| | 13 | 43 | 32 | 29 95 | 29 92 | N. | Cloudy—some snow |
| | 14 | 49 | 37 | 29 86 | 29 84 | N. W. | Fair |
| | 15 | 52 | 37 | 29 77 | 29 43 | S. W. | Sun and clouds |
| | 16 | 51 | 37 | 29 69 | 29 50 | S. E. | Clear, *a. m.*—small rain |
| | 17 | 54 | 41 | 29 69 | 29 67 | S. W. | Sun and showers |
| | 18 | 59 | 42 | 29 80 | 29 65 | S. | Fair |
| | 19 | 60 | 37 | 29 98 | 29 80 | S. | Fair |
| | 20 | 61 | 43 | 30 20 | 30 15 | S. E. | Fair |
| | 21 | 63 | 42 | 30 20 | 30 20 | W. | Fair |
| | 22 | 63 | 42 | 30 20 | 30 20 | W. | Fair |
| | 23 | 66 | 41 | 30 26 | 30 20 | W. | Fair |
| | 24 | 64 | 38 | 30 26 | 30 21 | S. E. | Fair |

| BAROMETER. | | | THERMOMETER. | | |
|---|---|---|---|---|---|
| Highest, April | 24 | 30.26 | Highest, April | 23 | 66 |
| Lowest, ditto | 6 | 29.33 | Lowest, ditto | 12 | 29 |
| Mean - - | 29 | 29.79½ | Mean - - - - - - | | 47½ |

### OBSERVATIONS.

March 30. *Cirro-Cumuli* observed in the sky in the morning.

April 6. The maximum of the Thermometer happened at 11 o'clock, *p. m.*

8. *Salices*, Vitellina, Fragilis, Rubra, Viminalis, Velutina, Russeliana, and others in flower

14. The modification of cloud, called *Cirro-Stratus*, observable in sky.

15. *Cirro-Cumuli* observed

16. *Cirri* in short tufts pointing to the South East, succeeded by rain.

17. *Cirro-Strati* in the sky.

18. *Cirro-Cumulus* prevails.

19. Very hazy moon.

20. The wryneck *Jynx Torquilla* first heard. I observed the swallow, *Hirundo Rustica*, as well as the martin, *Hirundo Urbica* flying about in the neighbourhood of West Ham.

21. Swallows first seen at Clapton.

22. *Cuckoo* first heard.

23 *Cirro-Strati* prevail.

24. *Cirro-Cumuli* observable.

By a Hygrometer of a peculiar construction which I make use of (see an account of it in Phil. Mag. for November 1801,) it appears that the air has been extremely dry ever since March 30th.

